# The impact of COVID-19 on clinical outcomes of burn patients

**DOI:** 10.1093/burnst/tkad042

**Published:** 2023-12-05

**Authors:** Elliot T Walters, Alen Palackic, Camila Franco-Mesa, Nikhil R Shah, Michael J Erickson, Steven E Wolf

**Affiliations:** Department of Surgery, University of Texas Medical Branch, 301 University, Galveston, TX, USA; Department of Surgery, University of Texas Medical Branch, 301 University, Galveston, TX, USA; Division of Plastic, Aesthetic and Reconstructive Surgery, Department of Surgery, Medical University of Graz, Graz, Austria; Department of Surgery, University of Texas Medical Branch, 301 University, Galveston, TX, USA; Department of Surgery, University of Texas Medical Branch, 301 University, Galveston, TX, USA; Department of Surgery, University of Texas Medical Branch, 301 University, Galveston, TX, USA; Department of Surgery, University of Texas Medical Branch, 301 University, Galveston, TX, USA

**Keywords:** COVID-19, infection, mortality, burn, thrombosis, scarring

## Abstract

**Background:**

Multiple studies have shown the SARS-CoV-2 virus (COVID-19) to be associated with deleterious outcomes in a wide range of patients. The impact of COVID-19 has not been well investigated among burned patients. We suspect that patients will have worsened respiratory and thrombotic complications, ultimately leading to increased mortality. The objective of this study is to determine the impact a concurrent infection of COVID-19 has on clinical outcomes after a burn injury.

**Methods:**

This is a retrospective, propensity matched, cohort study. We examined a de-identified database of electronic medical records of over 75 million patients across 75 health care associations in the United States for patients treated for thermal burns from 1 January 2020, to 31 July 2021, and those who also were diagnosed with COVID-19 infection within one day before or after injury based on International Classification of Disease, tenth revision (ICD-10) codes. Study participants included adults who were treated for a burn injury during the study period.

**Results:**

We included 736 patients with burn injury and concomitant COVID-19 infection matched to 736 patients with burn injury and no concurrent COVID-19 infection (total 1472 patients, mean age 36.3 ± 24.3). We found no significant increase in mortality observed for patients with concurrent COVID-19 (OR 1.203, 95% CI 0.517–2.803; *p* = 0.6675). We did observe significant increase in infections (OR 3.537, 95% CI 2.798–4.471; *p* = 0.0001), thrombotic complications (OR 2.342, 95% CI 1.351–4.058; *p* = 0.0018), as was the incidence of hypertrophic scarring (OR 3.368, 95% CI 2.326–4.877; *p* = 0.0001).

**Conclusions:**

We observed that concurrent COVID-19 infection was associated with an increase in infections, thrombosis and hypertrophic scarring but no increase in mortality in our cohort of burn patients.

Highlights

- The impact of COVID-19 has on clinical outcomes after burn injury was examined.
- We found a higher incidence of mortality, infectious, thrombotic and wound healing complications among burn patients with a concurrent COVID-19 infection.
- Patients experiencing a COVID-19 infection during the acute phase of burn care are at increased risk of morbidity.

## Background

Since the beginning of the coronavirus 2019 pandemic, efforts have been focused on understanding the physiologic changes associated with the disease [1, 2]. It is now well known that COVID-19 infection causes a dysregulated inflammatory state, which occasionally leads to severe thrombotic and hemodynamic consequences [2, 3]. This response is particularly strong in patients with advanced symptoms [3]. Similarly, burn patients present with a hypermetabolic and hypercytokinetic state secondary to the injury [4]. With over 32 million COVID-19 cases and 11 million burn patients per year worldwide, it is not surprising to encounter burn patients with an active COVID-19 infection [5]. Emerging literature suggests that concomitant affliction with both conditions amplifies the overall inflammatory response, thus increasing the risk of mortality and comorbidities for these patients [4, 6, 7]. However, to the best of our knowledge, no broad-based data is extant describing outcomes of burn patients with active COVID-19 infection [4]. We suspect that patients will have worsened respiratory and thrombotic complications leading to worsened morbidity and mortality. The purpose of this study is to evaluate short-term mortality and other outcomes of burn patients with concomitant COVID-19 compared to matched COVID-19 negative counterparts.

## Methods

### Data source

The TriNetX database (Cambridge, MA) is a federated health research network providing access to aggregated counts and statistical summaries from the electronic medical records of over 75 million patients and across 75 health care organizations (HCO’s). This database allows us to examine patient diagnoses, procedures, laboratory values and treatments throughout their treatment course; both patient and the HCO’s data sources remain anonymous therefore we cannot provide information on specific patients or the HCO’s. Participating HCO’s are typically large academic centers comprising of main and satellite hospitals and outpatient clinics with a mix of inpatient, outpatient and specialty services. TriNetX is HIPAA and GDPR compliant and the study does not use Protected Health Information (PHI). Analysis of the data was done upon information available in the platform at the time of inquiry. These data are continually updated, and are available form the data source. This study was therefore pre-approved by the IRB committee at the University of Texas Medical Branch, Texas. Data was collected on 12 December 2022.

### Patient selection

**Figure 1:**
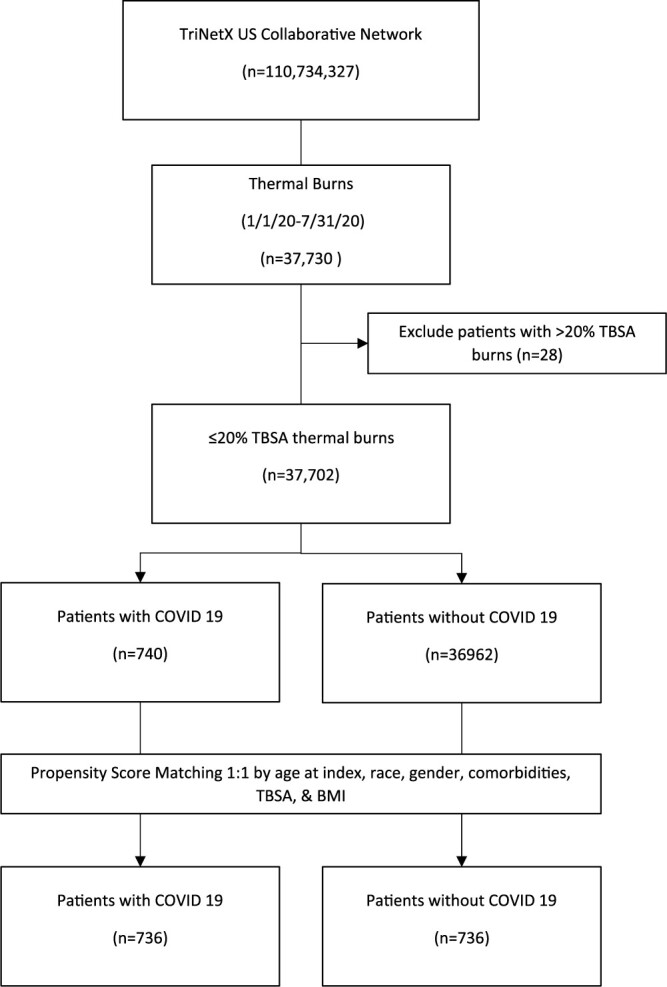
Flow chart of cohort construction. *TBSA* total body surface area

From this database we extracted all adult patients (>18 years old) who experienced a burn (ICD-10 codes T20–T25, T30–T34) between 1 January 2020 (just prior to the first diagnosed case of COVID-19 in the United States) and 31 July 2021, this produced a cohort of 37,730 patients. When evaluating our patient cohort we discovered that the majority of patients within the cohort had only small burns (<20%). We further censored out any patient with a burn equal to, or greater than, 20% TBSA. A flow chart of cohort construction can be seen in Figure 1. From this cohort we identified 740 persons also diagnosed with COVID-19 (ICD-10 code U07.1) within 1 day before or after their burn injury and 36,962 without a diagnosis of COVID-19 within 1 day before or after their burn injury. As the large disparity in cohort size introduced significant differences between cohort composition, propensity matching was employed to extract two cohorts of similar size and composition from our overall data. For analysis of outcomes, patients from the larger cohort were matched to patients from the smaller cohort based on age at time of injury, comorbidities, burn size and location.

### Statistical analysis

All data analysis was performed within the TriNetX platform. To protect PHI all patient counts <10 were rounded up to 10. This may influence measures of association for small patient counts. Patient characteristics were described by percentages, means and standard deviations. The TriNetX platform performed a 1:1 propensity score match using a greedy, nearest neighbor matching algorithm to account for confounding variables. Propensity score matching (PSM) is a common statistical matching technique used to evaluate all patients within a study by accounting for covariates that may act as confounders such as comorbidities, demographics, or burn size. Specifically, for our study, we included: age at time of burn, burn TBSA, burn location and comorbidities. Potential bias is reduced by assigning a propensity score to each patient. This is done by estimating the predicted probability of group membership while accounting for these confounders through regression techniques. Patients from each cohort are then matched based on these scores to produce two cohorts of similar size and characteristics; those who did not match were censored from the final analysis. TriNetX uses a caliper of 0.1 pooled standard deviations of the propensity scores in aggregate. As a result, COVID-19 positive patients with very different scores are likely ‘unique’ in that they didn’t have a matching counterpart in the COVID-19 negative group and are thus excluded from the analysis cohort. Unfortunately, due to the blinded nature of this database we cannot identify which characteristics contributedto the patients ‘uniqueness’.

Outcomes analyzed were based on ICD-10 definitions of mortality, prothrombotic outcomes (venous thromboembolism, pulmonary embolism and arterial thromboembolism), infection (bacterial, viral, fungal), respiratory failure, organ failure (heart, kidney and liver), wound healing and scarring (hypertrophic). Procedures evaluated included surgical excision, autografting, intubation and ventilator management based on CPT code definitions and included all outcomes from 30 days post-injury until the date of data collection (12 December 2022). Corresponding codes for these outcomes and procedures can be found in Table 1.

**Table 1:** Billing codes for procedures and diagnoses

**ICD-10 CM code for diagnoses**
Covid-19: U07.1
Burn injury: T20–25, T30–34 Inhalation injury: J70.5
**CPT codes for procedures**
Autografting: 15100, 15120
Intubation: 447996002, 52765003, 31500
Surgical excision: 15002, 15004
Surgical excision >100 cm^2^ or > 1% TBSA: 15003, 15004
Ventilator management: 1015098
**ICD-10 CM codes for outcomes**
Acute kidney injury: N17
Acute respiratory distress syndrome: J80
Death: R99, R69
Heart failure: I50
Hepatic failure or fibrosis: K72, K74
Hypertrophic scarring: L91
Infection (all): A00-B99
Infection (bacterial, soft tissue): A49, B95, B96, A48.8
MRSA infection: B95.62, A49.02, A41.02, J15.212. B95.7
Pneumonia (bacterial and viral): J10, J12, J15, J16, J18
Pseudomonas infection: B96.5, J15.1
Respiratory failure: J96
Sepsis: A40, A41, R65
Thrombosis (pooled): (deep vein, arterial, pulmonary): I26, I74, I82
Thrombosis (deep vein): I82
Thrombosis (arterial): I74
Thrombosis (pulmonary): I26

Due to the aggregated nature of this database, certain aspects of interest could not be measured directly. The code for ventilator management was used to determine the number of patients intubated and instances of this code per patient were used to determine ventilator days. As all patients receiving surgical excision were coded with CPT 15002 or 15004 (first 100cm^2^ or 1% TBSA), it was assumed that any patient coded with 15003 or 15005 (each additional 100cm^2^ of 1% TBSA) had excision beyond 100cm^2^ or 1% TBSA and therefore a more extensive excision.

Statistical analysis was performed using the TriNetX analysis tool package. Baseline comparison statistics analysis was applied to describe the patient demographic information. Measures of association for outcome analysis including *p*-value and odds ratio with 95% CI using *z*-test were performed. All data was compiled in Microsoft Excel (Redmond, WA).

## Results

Prior to propensity matching we found 36,962 burned patients without a diagnosis of COVID-19 and 740 burn patients with a confirmed diagnosis of COVID-19. After propensity score matching, 736 patients were included in each group for a total combined cohort of 1472. Unmatched demographic and comorbidity data can be found in Tables 2 and 3. Outcome results for matched patients are reported in Tables 4 and 5 and discussed within the text of this manuscript. The average age for patients with COVID-19 was 36.3 ± 24.3 and 35.2 ± 24.2 for those without COVID-19. We included 438 (59.5%) COVID-19 positive men and 433 (58.8%) men without COVID-19. Women included 296 (40.2%) and 298 (40.5%) of each group, respectively. The ethnic distribution of patients among the COVID-19 cohort was 61.6% (453) white, 16.4% (121) black, 14.5% (107) Hispanic or Latino, 2.5% (18) Asian, 1.4% (10) native American, and 1.4% (10) native Hawaiian or other Pacific Islander. Burn patients without COVID-19 were similarly matched according to ethnicity as shown in Table 2. Comorbidity data can be found in Table 3 and shows similar distribution of common comorbidities between groups.

**Table 2:** Patient demographics

	**Prior to matching**			**After matching**		
**Feature**	**COVID +** **(n = 740)**	**COVID –** **(n = 36 222)**	***P***	**COVID +** **(n = 736)**	**COVID –(n = 736)**	***P***
Age, y	36.4 ± 24.3	31.4 ± 23.2	0.0001	36.3 ± 24.3	36.2 ± 24.2	0.9538
Male sex, n (%)	442 (59.73)	21 466 (59.262)	0.7978	438 (59.511)	433 (58.832)	0.7909
Female sex, n (%)	296 (40)	14 513 (40.067)	0.9707	296 (40.217)	298 (40.489)	0.9154
Ethnicity, n (%)						
White/Caucasian	455 (61.486)	21 952 (60.604)	0.6267	453 (61.549)	452 (61.413)	0.9573
Black	122 (16.486)	6687 (18.461)	0.1701	121 (14.538)	117 (15.897)	0.777
Hispanic/Latino	107 (14.459)	4132 (11.407)	0.0099	107 (14.538)	123 (16.712)	0.2507
Asian	18 (1.486)	733 (2.024)	0.4352	18 (2.446)	14 (1.902)	0.4747
Native American	11 (1.486)	334 (0.922)	0.114	10 (1.359)	10 (1.359)	1
Hawaiian/ Pacific Islander	10 (1.351)	87 (0.24)	0.0001	10 (1.359)	10 (1.359)	1
BMI	26.6 ± 8.27	25.6 ± 7.89	0.0169	26.6 ± 8.29	26.7 ± 7.91	0.841

**Table 3:** Patient comorbidities

	**Prior to matching**			**After matching**		
**Comorbidity**	**COVID +** **(n = 740)**	**COVID –** **(n = 36 222)**	***P***	**COVID +** **(n = 736)**	**COVID –** **(n = 736)**	***P***
	
Hypertension, n (%)	180 (24.324)	4366 (12.053)	0.0001	176 (23.913)	178 (24.185)	0.9029
Chronic lower lung disease, n (%)	115 (15.541)	3596 (9.928)	0.0001	112 (15.217)	106 (16.712)	0.6597
Diabetes mellitus, n (%)	134 (18.108)	2339 (6.457)	0.0001	130 (15.217)	123 (16.712)	0.6287
Ischemic heart disease, n (%)	76 (10.27)	1447 (3.995)	0.0001	73 (9.918)	66 (8.967)	0.5327
Chronic kidney disease, n (%)	40 (5.405)	899 (2.482)	0.0001	40 (5.435)	48 (6.522)	0.3791
Heart failure, n (%)	53 (7.162)	867 (2.394)	0.0001	49 (6.658)	50 (6.793)	0.9171
Cerebrovascular disease, n (%)	68 (0.189)	1068 (2.948)	0.0001	64 (8.696)	64 (8.696)	1
Nicotine dependence, n (%)	140 (18.919)	3975 (10.974)	0.0001	136 (18.478)	138 (16.712)	0.8935
Alcohol-related disorders, n (%)	70 (9.459)	1516 (4.185)	0.0001	67 (9.103)	66 (8.967)	0.9276

**Table 4:** Patient outcomes post-matching

**Outcome**	**COVID + (n = 736)**	**COVID – (n = 736)**	**Odds ratio (OR)**	**Confidence Interval (CI)**	***P***
Mortality, % (n)	1.6 (12)	1.4 (10)	1.203	0.517–2.803	0.6675
Infection, % (n)	45.4 (334)	19.0 (140)	3.537	2.798–4.471	0.0001
Sepsis, % (n)	5.4 (40)	2.7 (20)	2.057	1.191–3.555	0.0084
Pseudomonas infection, % (n)	1.4 (10)	1.4 (10)	—	—	1
MRSA infection, % (n)	3.4 (25)	1.4 (10)	2.553	1.217–5.354	0.0103
Pneumonia, % (n)	8.3 (61)	5.4 (40)	1.572	1.041–2.376	0.0304
Respiratory failure, % (n)	6.8 (50)	4.2 (31)	1.658	1.046–2.626	0.0299
Acute respiratory distress syndrome, % (n)	1.4 (10)	1.4 (10)	—	—	1
Acute kidney injury, % (n)	5.2 (38)	4.6 (34)	1.124	0.699–1.806	0.6288
Heart failure, % (n)	1.8 (13)	1.4 (10)	1.32	0.575–3.031	0.5118
Hepatic failure, % (n)	1.4 (10)	1.4 (10)	—	—	1
Thrombosis (deep vein, arterial, pulmonary embolism), % (n)	5.8 (43)	2.6 (19)	2.342	1.351–4.058	0.0018
Thrombosis (deep vein), % (n)	4.5 (33)	1.6 (12)	2.832	1.451–5.528	0.0015
Thrombosis (arterial), % (n)	1.4 (10)	1.4 (10)	—	—	1
Thrombosis (pulmonary embolism), % (n)	1.8 (13)	1.4 (10)	1.305	0.569–2.996	0.5284
Hypertrophic scarring, % (n)	16.6 (122)	5.6 (41)	3.368	2.326–4.877	0.0001

**Table 5:** Procedural interventions

**Surgery/Procedure**	**COVID + (n = 736)**	**COVID –(n = 736)**	**Odds ratio (OR)**	**Confidence interval (CI)**	***P***
Surgical excision, % (n)	18.5 (136)	8.6 (63)	2.421	1.761–3.329	0.0001
Extensive excision, % (n)	13.6 (100)	6.7 (49)	2.204	1.54–3.155	0.0001
Autografting, % (n)	16.2 (119)	6.8 (50)	2.646	1.869–3.747	0.0001
Intubation, % (n)	1.4 (10)	1.4 (10)	—	—	1
Median ventilator days, (n)	4	3	—	—	0.8889

Our primary outcome was mortality which showed no significant difference among COVID-19 positive burn patients (1.6% *vs* 1.4%, OR 1.203, 95% CI 0.517–2.803, *p* = 0.6657).

Infections of all types (bacterial, viral and fungal, deep and superficial) were more common (45.4% *vs* 19.0%, OR 3.537, 95% CI 2.798–4.471, *p* < 0.0001) among the burned COVID-19 positive patients. Bacterial infections were especially more frequent (8.0% *vs* 4.2%, OR 1.982, 95% CI 1.267–3.1, *p* = 0.0023). Not surprisingly we found a higher incidence of sepsis (5.4% *vs* 2.7%, OR 2.057, 95% CI 1.191–3.555, *p* = 0.0084) and pneumonia (8.3% *vs* 5.4%, OR 1.572, 95% CI 1.041–2.376, *p* = 0.0304) in this group. Respiratory failure was significantly more likely among burned COVID-19 positive patients (6.8% *vs* 4.2%, OR 1.658 95% CI 1.046–2.626, *p* = 0.0299). Additionally, we found an increased incidence of intubation (6.4% *vs* 1.9%, OR 3.471 95% CI 1.967–6.123, *p* < 0.0001) but not ventilator days (median 6 *vs* 3.5, *p* = 0.2434). Interestingly, acute respiratory distress syndrome (ARDS) was uncommon in this overall patient cohort (1.4%* vs* 1.4% *p* = 1.000) which did not allow for any statistical comparison.

When organ failure was examined, it was noted that the heart and kidneys were more likely to be affected in COVID-19 positive burned patients. COVID-19 positive burn patients were not more likely to experience acute kidney failure (5.2% *vs* 4.6%, OR 1.124, 95% CI 0.699–1.806, *p* = 0.6288). Heart failure also appeared to be rare among this group of patients (1.8% *vs* 1.4%, OR 1.32, 95% CI 0.575–3.031 *p* = 0.5118). The liver also appeared to be spared as liver failure or fibrosis was uncommon (1.4% *vs* 1.4% *p* = 1.000) in both cohorts.

In regard to thrombotic complications, we found an increased rate in pooled thrombotic complications (deep venous thrombosis, arterial thrombosis and pulmonary embolism) (5.8% *vs* 2.6%, OR 2.342, 95% CI 1.351–4.058, *p* = 0.0018). This appears to be driven largely by deep venous thrombosis (4.5% *vs* 1.6%, OR 2.832, 95% CI 1.451–5.528, *p* = 0.0015) as arterial thrombosis (1.4% *vs* 1.4%, *p* = 1) and pulmonary embolism (1.8% *vs* 1.4%, OR 1.305, 95% CI 0.569–2.996 *p* = 0.5284) was rare among both cohorts. Laboratory values of thromboelastography (TEG), partial thromboplastin time (PTT) or international normalized ratio (INR) were not available in our dataset to fully characterize the coagulation profile.

Pertaining to wound healing and scarring, burned patients with COVID-19 were not only more likely to receive surgical excision (18.5% *vs* 8.6% OR 2.421, 95% CI 1.761–3.329, *p* = 0.0001) but also to receive extensive excision (13.6% *vs* 6.7% OR 2.204, 95% CI 1.540–3.155, *p* = 0.0001) despite being matched for burn size. COVID-19 positive patients also were more likely to receive autografting (16.2% *vs* 6.8% OR 2.646, 95% CI 1.869–3.747, *p* = 0.0001). From the data it is not clear if this increased autografting was due to prior graft failure or primary grafting of wounds too extensive to heal from conservative measures alone. Furthermore, an increased incidence of hypertrophic scarring (16.6% *vs* 5.6%, OR 3.368, 95% CI 2.326–4.877, *p* = 0.0001) was noted among this patient group (Tables 4 and 5).

## Discussion

Even though the novel coronavirus COVID-19 (SARS-CoV-2) has impacted patients and providers at all levels for over two years, it is only now that the clinical implications of the virus are beginning to be understood. Despite the extraordinary measures implemented to control this disease it has continued to spread throughout our communities. Over the last several decades improvements in fire safety and burn care have reduced the frequency and severity of burn injuries. However, the COVID-19 lockdown raised several concerns that affected the burn patient population. The prolonged stay in confined areas attributed to quarantining and the community’s fear of contracting the virus when seeking healthcare could lead patients to present with delayed burn injuries [8–10]. Despite this, little literature is available investigating how COVID-19 positivity impacts the burn patient. We present the first large scale, multi-institutional study of clinical outcomes in burned patients with a concomitant diagnosis of COVID-19.

### Mortality

Almost all variables were statistically significant prior to propensity score matching, including mortality. COVID-19 causes immune dysregulation, likewise, burn injuries generate a similar cytokinetic storm [11–13]. Consequently, the ‘two-hit hypothesis’ of concomitant burn injury and COVID-19 infection would lead to an overwhelming inflammatory response. If this were the case, we would expect to see a large increase in mortality among burn patients. However, after matching for age, comorbidities, and burn size our data failed to show a statistically significant increase in mortality. While our data does not evidence a drastic increase in mortality this might be explained by the fact that the common pathway of interleukin (IL)-6, IL-8 and tumor necrosis factor α (TNF-α) activation are already activated by the burn injury thus overshadowing the COVID-19 cytokinetic dysregulation of these cytokinetic pathways. Further investigation is required as more data becomes available.

### Thrombosis

Burned patients often have mobility difficulties and may undergo several procedures. Unsurprisingly, Virchow’s triad of stasis, endothelial injury and hypercoagulability are satisfied in most burned patients. For these reasons we expect to see elevated rates of thrombotic complications. However, numerous studies have shown a low incidence of VTE [14–16]. These studies suggest that these events are rare due to the hyperdynamic circulatory state and increased blood flow to burned areas [17]. Thus, only high-risk patients need VTE prophylaxis including those with large burns or of advanced age [16, 18, 19]. These guidelines predate COVID-19 and we have shown that, despite the decreased pro-thrombotic activity in normal burn patients, concomitant infection with COVID-19 may warrant special consideration.

The etiology of thrombotic complications in patients with COVID-19 was well described in a review by Labò et al. [11]. Angiotensin Converting Enzyme II (ACEII), which is a key entry point for SARS-CoV-2 virus, is prominently expressed on the endothelial lining of blood vessels. Once through, access is freely granted to the cell leading to viral proliferation, lysis and exposure of the prothrombotic basement membrane. Likewise, viral binding causes internalization of ACEII which results in increased plasma concentration of angiotensin II. Elevated angiotensin II assists in vasoconstriction and a pro-thrombotic, proinflammatory state. Circulating concentrations of IL-6 are increased by this action which further enhances the pro-thrombotic state. Of note, SARS-CoV-2 can also bind to, and activate, TNF-α converting enzyme (TACE/ADAM17). This enzyme is responsible for the increase of active circulating TNF-α. Combined with IL-6 and other activated cytokines, these act as chemo-attractants to monocytes and macrophages. The outcome being localized vasculitis secondary to massive activation of immune cells within the vessel wall.

Not surprisingly, COVID-19 positive burned patients were 2.641 times more at risk of thrombotic complications over their matched counterparts. While the function of ACEII has not been shown to affect the physiology of the burn patient, a significant overlap exists in the activity of IL-6 and TNF-α. These factors, in addition to the epithelial damage caused by the virus, may offset any antithrombotic state present in the burn patient. Our data failed to identify an increased risk of acute renal failure and heart failure but other studies of SARS-CoV-2 have identified such a risk and suggests this may be related to microthromboses [20]. If this is the case this may suggest an additional benefit from VTE chemoprophylaxis while admitted and as soon as reasonably safe and appropriate.

### Infection

Lachiewicz reported that patients with severe burns have diminished immunity leading to more frequent and severe bacterial, viral and fungal infections [21]. This is, in part, due to an overwhelming influx of circulating cytokines. Studies have shown patients suffering from COVID-19 also develop leukopenia leading to compromised immunity [12]. While respiratory infections and complications were modestly increased in our cohort, bacterial soft tissue infections were also increased suggesting that the immune dysfunction from burn injury and COVID-19 infection is compounded, particularly affecting the already damaged and vulnerable integumentary system. In this regard, our data demonstrated a significantly higher association with diagnosis of sepsis (OR: 3.696) and pneumonia (OR: 4.141). Interestingly, ARDS from all causes was so rare that data analysis was not possible.

### Wound healing and scarring

In addition to increased soft tissue infections, we also identified an increase in surgical excision and autografting and report a 3.368 times increase in hypertrophic scarring. Since all patients were matched for burn size and location it is not clear from our data what factors lead to this increase in procedures. Lee et al., in a 2018 review on the pathophysiology of hypertrophic scar formation, detailed the crucial roles IL-6, IL-8 and TNF-α play throughout the wound healing process [22]. IL-6 is active during the proliferation phase and thought to be pro-fibrotic, IL-8 is a powerful stimulator of angiogenesis, and TNF-α increases matrix metalloproteinases within the tissue bed [22]. This combined effect leads to increased fibrinogenesis and collagen deposition. Since SARS-CoV-2 has also been shown to upregulate these cytokines, it is likely the increased circulation leads to a highly active or prolonged inflammatory phase and/or proliferative phase of healing.

### Limitations

The findings of this study should serve as a starting point for further investigation in management of patients with concomitant COVID-19 and burn injuries. Despite a large cohort, potential limitations are present. This data is derived from a large, aggregated, prospectively maintained database and as such specific patient medical records are not available for review, nor can we determine if patients were treated at an ABA certified burn center. Additionally, only limited follow up is available for many patients making any kind of long-term outcome analysis impossible. Due to the retrospective nature of this study, we were unable to show direct causality; this is especially relevant in considering the number of procedures received among our cohorts. When more data becomes available further investigation will be needed to determine the effect of COVID-19 on this patient population. Despite the limitations, the findings of this study will provide the groundwork for further investigations in the management of burned patients with concomitant COVID-19 infection.

## Conclusions

To our knowledge, this is the first large, multicenter study that evaluates the outcomes of burn patients with a concomitant diagnosis of COVID-19. We presented the results of a propensity matched cohort of 482 burn patients with and without COVID-19. While we found no statistically significant increase in mortality among these patients, an increased risk of infection, scarring and thrombotic complications was identified. It is clear this novel virus exerts many clinically significant effects on baseline burn physiology in the adult population, however the current knowledge gap remains vast. As more data becomes available further studies should examine long-term outcomes and treatment modalities that will help to mitigate the deleterious effects of this new disease.

## Declarations

TriNetX is HIPAA and GDPR compliant and the study does not use Protected Health Information (PHI). This study was pre-approved by the ethics and IRB committee at the University of Texas Medical Branch, Texas.
